# The Developmental Trajectory of Intramaze and Extramaze Landmark Biases in Spatial Navigation: An Unexpected Journey

**DOI:** 10.1037/a0039054

**Published:** 2015-04-06

**Authors:** Matthew G. Buckley, Mark Haselgrove, Alastair D. Smith

**Affiliations:** 1 School of Psychology, University of Nottingham

**Keywords:** spatial navigation, learning, development, local, distal, landmark

## Abstract

Adults learning to navigate to a hidden goal within an enclosed space have been found to prefer information provided by the distal cues of an environment, as opposed to proximal landmarks within the environment. Studies with children, however, have shown that 5- or 7-year-olds do not display any preference toward distal or proximal cues during navigation. This suggests that a bias toward learning about distal cues occurs somewhere between the age of 7 years and adulthood. We recruited 5- to 11-year-old children and an adult sample to explore the developmental profile of this putative change. Across a series of 3 experiments, participants were required to navigate to a hidden goal in a virtual environment, the location of which was signaled by both extramaze and intramaze landmark cues. During testing, these cues were placed into conflict to assess the search preferences of participants. Consistent with previously reported findings, adults were biased toward using extramaze information. However, analysis of the data from children, which incorporated age as a continuous variable, suggested that older children in our sample were, in fact, biased toward using the intramaze landmark in our task. These findings suggest the bias toward using distal cues in spatial navigation, frequently displayed by adults, may be a comparatively late developing trait, and one that could supersede an initial developmental preference for proximal landmarks.

Learning the location of important places in the world is a fundamental ability for humans and nonhuman animals alike. Accordingly, the study of the mechanisms underlying spatial navigation has been a focus for many fields in the behavioral sciences. Navigation is subserved by a variety of processes, from the moment-to-moment updating of position through movement kinematics (i.e., path integration; Loomis et al., 1993) through to enduring long-term representations of places and landmarks in temporal cortices (e.g., the parahippocampal place area; Epstein & Kanwisher, 1998). Recently, there has been particular debate about the visual properties of the world that are used to encode and represent an environment (see Pearce, 2009; Jeffery, 2010). These properties have been broadly split into two domains: *distal information* that is provided by the boundary walls of an environment, which may be orientated by landmark cues (e.g., Bullens et al., 2010; Doeller & Burgess, 2008); and *proximal* information that is provided by landmarks that are close to a goal location (e.g., Wilson & Alexander, 2008), or beacons that are located at a goal location (e.g., Redhead & Hamilton, 2009).

Both distal and proximal information are commonly used to support navigational behavior; however, experiments in which the boundary walls of the environment have provided geometric information have tended to reveal biases toward distal information. For example, some studies of reorientation behavior have demonstrated a reliance on the geometric information provided by the walls of the enclosure, at the expense of information provided by proximal landmark cues within the arena. This has been observed in adults (Redhead & Hamilton, 2007, 2009), children (e.g., Lee & Spelke, 2008, 2010), rats (e.g., Graham, Good, McGregor, & Pearce, 2006; Wall, Botly, Black, & Shettleworth, 2004), and pigeons (Kelly, Spetch, & Heth, 1998). Under other circumstances, however, participants are able to use proximal information, in combination with distal cues, to reorient more accurately. For example, children can successfully integrate landmarks and geometric cues (i.e., overcome a geometric bias) when they are tested in larger spaces (e.g., Learmonth, Nadel, & Newcombe, 2002; see Cheng, Huttenlocher, & Newcombe, 2013, for a review).

In contrast to studies of reorientation, a different pattern of biases has been observed in place learning experiments, where circular boundary walls are orientated by distal landmark cues. Here, experiments conducted with children, using a variety of different paradigms, have shown that navigation based on proximal landmarks predominates in childhood, at the expense of distal landmarks. For example, Laurance, Learmonth, Nadel, and Jacobs (2003) tested children in a virtual environment, requiring them to navigate to a goal location within a circular arena that was housed in a large square room. To orient the circular arena, the walls of the square room contained pictures of everyday objects that were visible beyond the circular wall. Initially, the goal was visible to participants, although its location did not remain in a constant position within the environment. During this stage of the experiment, groups of 5- to 10-year-old children navigated to the goal as efficiently as adult participants, thus demonstrating effective learning about a beacon in the virtual arena. In the second stage of the experiment, the goal location was invisible to participants, but remained in a constant position. Consequently, to find the goal participants were required to navigate using the information provided by the distal picture landmarks that orientated the circular walls. Under these circumstances, there was a stepwise progression, with age, in efficiency to find the goal. The 5-year-old children took the longest to navigate to the goal, and latencies to find the goal decreased in older age groups, with the group of 9- and 10-year-old children finding the hidden goal in the same time as adults. These results suggest that young children are less able to navigate using distal landmark cues. It should be noted, however, that children were never required to find a hidden goal on the basis of just the distal landmark information, before being tested with a visible goal in the presence of the same distal cues that were not relevant to finding the goal. It remains unclear, therefore, whether the observed asymmetry was a consequence of differences in the processing of different navigational cues, or of testing order.

In real-world assessments of children’s navigation (i.e., those that require egocentric movement through a laboratory space rather than a virtual task), similar differences have been observed in the trajectories of learning about proximal and distal landmark cues: Children’s ability to navigate with intramaze landmarks appears to develop before they are able to navigate with extramaze landmark information (see Lehnung et al., 1998; Lehnung et al., 2003; Leplow et al., 2003; Overman, Pate, Moore, & Peuster, 1996). For example, Lehnung et al. (1998) introduced children to a circular arena that contained two intramaze landmarks and that was surrounded by four extramaze landmarks on the walls. The floor of the arena contained an array of lights, some of which played a tone when pressed (baited lights), while the rest of lights did not (unbaited lights). Children were required to navigate to the baited lights (the number of which was dependent on the age of the child), and were given training until they could locate the baited lights, without visiting unbaited lights, on two successive trials. Following acquisition of the task, a test was administered in which the intramaze landmarks were removed, thus, assessing learning relative to the four extramaze cues. Although the group of 10-year-old children could accurately navigate to the baited light points in the absence of the intramaze landmark cues, the 5-year-old group of children was disorientated, visiting many unbaited lights. Performance in the 7-year-old group was split: Half of the sample navigated accurately on the basis of the remaining extramaze cues and half, like the 5-year-old group, visited unbaited locations.

That young children preferentially navigate on the basis of proximal landmark cues is particularly interesting given that adults have been consistently found to show the opposite bias; namely, a preference for navigating on the basis of distal information, over proximal landmark cues. In an experiment conducted by Doeller and Burgess (2008), for example, participants in a compound group were required to collect an object within a circular virtual environment that contained an intramaze landmark, and that was always orientated by distal cues. Having collected the object, participants were asked to replace it, and the distance error between the replacement and its original location provided a measure of object−place memory. Following a series of training trials, participants in the compound group were given one of two test phases. For half of the participants, the circular boundary was removed and the objects had to be replaced by reference to just the intramaze landmark, which remained orientated by distal cues. For the other half, the intramaze landmark cue was removed and the objects had to be replaced with reference to just the circular boundary, which remained orientated by the distal cues. Performance in this compound group was compared with that of two control groups that performed the whole experiment with the orientation cues and either (a) just the intramaze landmark or (b) just the circular boundary. Participants in the compound group who were tested with the circular boundary showed equivalent performance to the boundary control group. However, participants in the compound group who were tested with the intramaze landmark displayed greater error compared with the landmark control group. In the parlance of associative learning theory, the presence of the circular boundary in the compound group *overshadowed*, or restricted, learning about the intramaze landmark; however, learning about the environmental boundary was immune to this effect (see also Doeller, King, & Burgess, 2008).

In a study explicitly designed to extend previous work with adults into the developmental domain, Bullens et al. (2010) adapted the design used by Doeller and Burgess (2008) to compare the navigational behavior of 5- and 7-year-old children with a group of adults. In their experiment, participants were led into a circular arena that contained a single intramaze landmark that was oriented by two extramaze landmarks located just beyond the circular walls. The placement of the intramaze landmark was manipulated during the course of the experiment, such that its position relative to the extramaze cues changed between blocks. On each trial, participants were required to remember the location of two pictures that the experimenter had hidden, separately, beneath one of the tiles that made up the floor. One picture remained in a fixed location relative to the extramaze cues, while the second picture was located at a fixed location relative to the intramaze landmark. Following disorientation, participants were presented with a duplicate of one of the hidden pictures and asked to place it on the floor where they thought that picture was hidden. Analysis of distance errors revealed that adults, in general, displayed greater accuracy than children and, in keeping with previous studies, were more accurate when searching for the picture that remained in a fixed location relative to the extramaze cues compared with the picture that remained in a fixed location relative to the intramaze landmark. Interestingly however, the 5- and 7-year-old children displayed similar levels of error for both pictures. Further inspection of the data revealed that adults were less accurate in relocating the picture that remained in a fixed location to the intramaze landmark due to a reliance on using the extramaze cues to guide their navigational behavior. In effect, adult participants ignored that the position of the intramaze landmark changed during the experiment, and replaced both pictures relative to the extramaze cues. Children, however, displayed no such bias.

It is worth noting that the distal cues in the experiment conducted by Doeller and Burgess (2008) and Bullens et al. (2010) should not be considered equivalent. In the virtual-navigation experiments conducted by Doeller and Burgess, the distal cues were projected at infinity and, as such, could not be used as positional cues when navigating to a target location. Instead, participants were required to use these distal cues to orient the circular wall, which did provide positional information. In contrast, the extramaze landmarks in the “real-world” experiments conducted by Bullens et al. were sufficiently close to the circular arena to provide positional information when navigating. Nevertheless, both experiments indexed place learning and, as we have discussed, both experiments found that adults preferentially navigate on the basis of distal information. The implication of previous navigational studies conducted with children, and especially that of Bullens et al. (2010), is that a bias toward using distal information to guide navigational behavior must develop at some point between the age of 7 and adulthood. As a result, the experiments we report here were designed to more closely characterize the nature of this developmental change. To do this, we adopted two particular approaches within our paradigm. The first was to use a developmental trajectory design, rather than the group-based approach that has been adopted by most of the studies assessing the development of proximal and distal cue use. The developmental trajectory approach offers certain advantages over traditional group designs, not least of which is that it permitted us to track the age-related development of any bias toward using extramaze cues within our sample, thus allowing a more exact identification of the age at which a switch toward using distal information might occur. Moreover, in the present context, a trajectory approach offered the opportunity to test a wider age range of children, rather than restricting testing to children who conformed to age groups defined on an a priori basis. In light of previous work, which has suggested that a bias toward using distal information might occur between age 7 and adulthood (see Bullens et al., 2010; Doeller & Burgess, 2008), we specifically recruited children 5 to 11 years of age (Experiments 1 and 2) and adults (Experiment 3).

Our second approach was to present environments within a virtual context, rather than testing within a real-world task involving full body movements. We did this because real-world comparisons of navigational behavior in children and adults can introduce confounds as a result of physical factors. In particular, the salience of both the intramaze and extramaze landmark cues will be influenced by the height of participants. As noted by Bullens et al. (2010), the intramaze landmark in their task was closer to the height of child participants than it was for adult participants. As such, it is possible that the intramaze landmark appeared more salient to children and, therefore, contributed to children’s reduced use of the extramaze landmarks relative to adult participants. Alternatively, the greater relative height of the circular walls for child participants compared with adult participants may have led children to focus more on the intramaze landmark than on the extramaze cues. Studying navigational behavior in virtual environments here allowed us to present a visual scene in which the relationship between the virtual eye height and experimental cues would be matched across all ages of participants. Importantly, we wanted to design our virtual environments to be comparable to the experiment conducted by Bullens et al. (2010), because it is their assessment of children that motivated the studies presented here. Consequently, we designed our environments to contain a single intramaze landmark, and we also presented extramaze landmarks that were located just beyond the circular walls of our arena.

In addition to assessing whether the age of children would affect their navigational performance, we also incorporated individual difference measures to explore whether additional factors could help point toward the underlying functional substrates of developmental change. Children in this study were tested as part of a larger scientific engagement event, which also administered measures of receptive vocabulary (British Picture Vocabulary Scale [BPVS III]; Dunn, Dunn, Styles, & Sewell, 2009), social behaviors relating to autism spectrum disorder (ASD; Social Aptitudes Scale [SAS]; Liddle, Batty, & Goodman, 2009), and behavioral traits relating to attention-deficit/hyperactivity disorder (ADHD; SWAN; Swanson et al., 2006). Although an in-depth discussion of how each of these factors might affect spatial navigational behaviors is beyond the scope of this article, there is good reason to predict that each of these factors may have a role to play within a typically developing population. For example, there have been suggestions that the combination of landmark and geometric cues in reorientation tasks is related to verbal ability (e.g., spatial grammar; Hermer-Vazquez, Moffet, & Munkholm, 2001; Hermer-Vazquez, Spelke, & Katsnelson, 1999; but see Bek, Blades, Siegal, & Varley, 2010; Ratliff & Newcombe, 2008). Equally, it has also been demonstrated that ASD is associated with suboptimal and nonsystematic search behavior in large-scale space (Pellicano et al., 2011). Finally, there is evidence that individuals with ADHD may demonstrate neglect-like inattention to stimuli presented in left hemispace (e.g., Bellgrove et al., 2008; Jones, Craver-Lemley, & Barrett, 2008), which could impact on encoding and exploration of space. As such, it is possible that children who score closer to clinically defined levels of ASD- and ADHD-like behavior may perform differently than other children (e.g., they may show a preference for a particular cue when other children do not, or they may perform less efficiently in general).

While previous studies have been based on examining performance after the removal of a cue that was present during the learning phase, or placing the two cues in conflict at test, there appears to be no published study that has compared the developmental trajectory of learning to extramaze cues with the trajectory of learning to intramaze landmarks. This is a fundamental starting point, because it can help to disentangle whether children are more likely to use one cue over the other because of proficiency (i.e., they are simply better able to navigate using one type of cue) or for other reasons (e.g., a differential weighting of cues if, and only if, they are presented together). A design that only addresses behavior when the two cues are presented in combination is unable to make this distinction and, as such, Experiment 1 was a between-participants design where an intramaze group was required to navigate to a hidden goal on the basis on an intramaze landmark, whereas an extramaze group was required to navigate to a hidden goal on the basis on extramaze landmarks.

## Experiment 1

Experiment 1 compared learning with either extramaze or intramaze cues in circumstances where just one of these cue types was presented during training and testing. During a series of acquisition trials, participants were required to navigate to a hidden goal, which remained in a constant position within a circular environment. For an extramaze group, the circle was orientated by four cues located just beyond the boundary of the arena, whereas, for an intramaze group, the circle contained a landmark that was located within the circular environment. Test trials were administered following the 12th, 16th, and 20th acquisition trials, during which the hidden goal was removed from the arena. Following the results of Laurance et al. (2003), we expected older children to learn the location of the goal more rapidly than younger children. Moreover, given that previous research has shown that the ability to navigate on the basis of proximal landmarks develops before the ability to navigate on the basis of distal information (e.g., Bullens et al., 2010; Lehnung et al., 1998), we also expected that navigation, measured either on the basis of the speed of acquisition or on the basis of performance at test, would be superior in the intramaze group than in the extramaze group for younger children, and vice versa for older children.

### Method

#### Participants

Forty-eight children (18 female), ages 74.04 (i.e., 6 years) to 135.48 (i.e., 11 years) months (*M* = 102.91 months, *SD* = 20.41) were recruited during Summer Scientist Week, an annual public engagement event conducted at the University of Nottingham (see http://www.summerscientist.org). All children had normal or corrected-to-normal vision, and participated with full parental consent. The age of participants in the extramaze group ranged from 74.04 months to 135.24 months, and the age of participants in the intramaze group ranged from 75.60 months to 135.48 months. The ages of male (*M* = 8.90 years, *SD* = 1.72) and female (*M* = 8.00 years, *SD* = 1.98) participants did not differ statistically in the extramaze group, *t*(22) = 1.20, *p* = .24, and, similarly, the ages of male (*M* = 8.59 years, *SD* = 1.52) and female (*M* = 8.54 years, *SD* = 1.81) participants in the intramaze group did not differ statistically, *t*(22) = .06, *p* = .95. Children were pseudorandomly assigned to one of the two experimental groups, with the constraint that the age range of the groups were closely matched, and were given a token that allowed them to play a fairground game at the event in return for participation. Measures of language ability (BPVS *M*_raw_ = 97.43, *SD* = 20.42), ASD (SAS *M*_total_ = 26.38, *SD* = 5.98), and ADHD (SWAN Inattentive subscale *M*_total_ = −6.57, *SD* = 10.12; SWAN Hyperactive−Impulsive subscale *M*_total_ = −8.49, *SD* = 9.88) were taken for these children.

#### Materials

All virtual environments were constructed, compiled, and displayed using MazeSuite software (Ayaz, Allen, Platek, & Onaral, 2008; see http://www.mazesuite.com). They were displayed full screen (33.17 × 20.73 cm) on an Apple Macbook Pro laptop computer running Windows 7. While conducting the experiment, a small table (approximately 60 cm in height) and accompanying chair were used to ensure the laptop was at the child’s height. An A4-sized (21.00 × 29.70 cm) piece of cardboard, with a hole cut out to reveal the cursor keys, was fixed over the keyboard during the experiment to ensure that participants did not press any additional keys.

**Figure 1 fig1:**
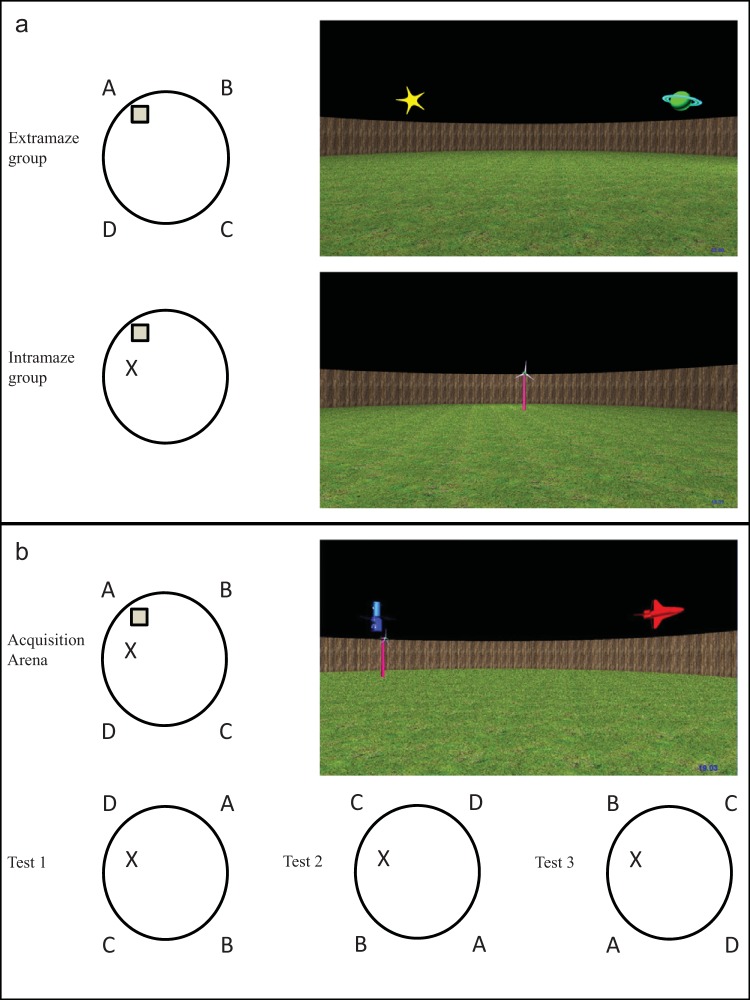
Letters A, B, C, and D represent the four extramaze cues, and letter X represents the intramaze landmark that was situated within the circular enclosure. The gray square indicates the position of the hidden goal. (a) Schematic diagrams and screen shots of the arenas used in Experiment 1 for the extramaze group (top left and right) and the intramaze group (bottom left and right). The test trial arenas in Experiment 1 were the same as the acquisition arenas, except for the removal of the hidden goal. (b) Schematic diagrams of the acquisition (top left) and test arenas (bottom left, center, and right) and a screen shot of the acquisition arena used in Experiments 2 and 3. See the online article for the color version of this figure.

Three virtual circular arenas were used in the experiment: an extramaze arena, an intramaze arena, and an instruction arena, all of which were viewed from a first-person perspective. A grass texture and a brown fence texture were applied to the floor and wall, respectively, of all arenas. Assuming a walking speed of 2 m/s, the diameter of the arenas was 12 m. All intra- and extramaze landmarks included in the experiment (see Figure 1) were imported into three-dimensional object modeling software (see http://www.blender.org), after which they were imported into MazeSuite. For the extramaze arena, four objects were located immediately beyond the circular wall of the arena and were placed equidistant from each other. The four extramaze cues were a planet, a star (see http://www.turbosquid.com), a space shuttle, and a model of the Hubble telescope (see http://www.nasa.gov). The intramaze arena contained a wind turbine (see http://www.turbosquid.com), which served as an intramaze landmark. Following a radial line of the circle, this landmark was located approximately 2.7 m from the center of the circle and, thus, 3.3 m from the circular wall. Finally, the instruction arena contained four extramaze cues and an intramaze landmark, although the identities of these objects differed from those presented in the extra- and intramaze groups. The four extramaze objects were a Legoman, a hot air balloon, a tower block, and a tree (see http://www.turbosquid.com), and the intramaze landmark was a model of an Apollo Lunar Module (see http://www.nasa.gov). Figure 1 shows the location of the hidden goal that, within all of the arenas, was a square-shaped region (2.12 m × 2.12 m, invisible to participants), the center of which was located 2.62 m from either the intramaze landmark (if present) or one of the extramaze objects (if present). Importantly, it was possible to navigate past the hidden goal area along each of its four sides. Consequently, participants could not, for example, simply traverse a path next to the circular boundary to find the hidden goal. Instead, participants were required to localize the target location with respect to the intra- and extramaze landmarks and the circular wall. Finally, both the intra- and extramaze arenas contained a flag that appeared at the goal location after 60 s of exploration on acquisition trials.[Fig-anchor fig1]

#### Design and procedure

This section will first describe the instructions that were given the participants, before outlining the procedural details of the experiment.

##### Instructions

Standardized verbal instructions were given by one experimenter to all participants, combined with a brief demonstration of the experiment. Here, we outline the verbal instructions in italics, and describe the demonstration in standard font. At the beginning of the experiment, participants were told the following story:

In this game we are trying to find William the Worm, who has been a sneaky worm and hidden under the grass in our garden. This means we can’t see where William is. To find him, what we have to do is walk around our garden, and when we step on his house William will pop up on the screen to let us know we’ve found him. Now, just like your house doesn’t move, neither does William’s. So the trick is to try and learn where William lives, because he’ll be hiding in the same place every go.

The experimenter then demonstrated two trials of the experiment in the instruction arena. At the beginning of the first demonstration trial, the experimenter began by rotating 360° in the center of the arena, and talked through the layout of the arena. *So, the garden is a big circle and we can’t walk past the fence. But behind the fence there are four things, a giant Legoman, a hot air balloon, a funny colored building, a tree* [said while rotating within the virtual environment to bring each object into view], *and in the middle of the garden there’s a moon-lander* [end of 360° rotation]. *Now, somewhere under the grass is William’s home* [at this point, the experimenter would take a meandering path around the arena], *but on the first go we don’t know where William is, so we just have to walk around and hope we step on his house. He’ll pop up on the screen when we do step on his house.*

Typically, the first instruction trial would last between 25 and 30 s. At the beginning of the second trial, the experimenter once again explained that William would always hide in the same place: *Now, the trick to the game is to remember where William was last time, because he’ll be hiding in the same place again.* The experimenter then walked in a direct route to the hidden goal, after which the MazeSuite application terminated. Before beginning the experimental task, children had the chance to ask questions, although all had grasped the concept of the game and were eager to try it themselves. Before the experimental trials began, the experimenter explained, *You use these keys to move around just like I did, the up arrow moves you forward, the down arrow moves you backward, and these sideways pointing arrows will turn you around* [said while pointing to the left and right cursor keys in turn].

If the time of any trial reached 55 s, the experimenter explained, *Now, if we don’t find William after a while he puts up a white flag to show us where he’s hiding. So if you see a white flag appear that’s William showing you where he is. Try to remember where he lives for the next go though, so we can find him without any help.*

##### Experiment

Participants sat not more than 50 cm from the screen. Presses on the “up” and “down” cursor keys permitted the participant to move forward and backward within the arena, respectively. Presses on the “left” and “right” cursor keys permitted the participant to rotate counterclockwise and clockwise within the arena, respectively. Participants were given 12 acquisition trials before receiving a test trial, which was followed by another four acquisition trials and a second test trial, after which there were another four acquisition trials followed by a third, and final, test.

Participants began each acquisition trial at the center of the circular arena; the direction in which participants faced at the start of the trial was randomized for every trial. There was no time limit for any trials, therefore, each trial ended only when the participant had navigated to the hidden goal zone. The hidden goal was deemed to be found as soon as participants traversed within any part of the square goal location. To aid participants in learning where the hidden goal was, a white flag appeared in the goal location after 60 s, and participants were required to navigate to the white flag to terminate the trial. Once the hidden goal had been found, a cartoon picture of a brown stripy worm, wearing sun glasses, appeared on screen. Superimposed over this image was the message, “Well done! You found William!” The next trial began automatically after this image had been displayed for 3 s.

During acquisition, the hidden goal remained in the same location on each trial, which was equidistant from both the intramaze landmark (intramaze group) and one of the four extramaze cues (extramaze group). The identity of the extramaze cue to which the hidden goal was closest was counterbalanced within the extramaze group, such that each of the four extramaze cues was closest to the hidden goal for six participants. For all test trials, the hidden goal zone was removed, and participants were allowed to search for 60 s having, again, begun in the center of the arena facing a random direction. At the end of the first and the second test trial, the message “Keep looking for William” was displayed for 3 s, after which the next block of four acquisition trials began automatically. The experimenter also gave this instruction verbally. At the end of the third test trial, the MazeSuite application terminated.

To assess navigational behavior over the course of the experiment, we recorded both the time taken to find the hidden goal and the length of the path traversed in virtual units (a measure that incorporates movement in the *x* and *z* planes, but does not include rotations around the *y* axis). The latency to find the goal is a common measure in studies of spatial navigation both in animals (e.g., Pearce, Roberts, & Good, 1998; Morris, 1981) and humans (e.g., Wilson & Alexander, 2008, 2010), as is path length (e.g., Bast, Wilson, Witter, & Morris, 2009; Redhead & Hamilton, 2007).

### Results

#### Acquisition

**Figure 2 fig2:**
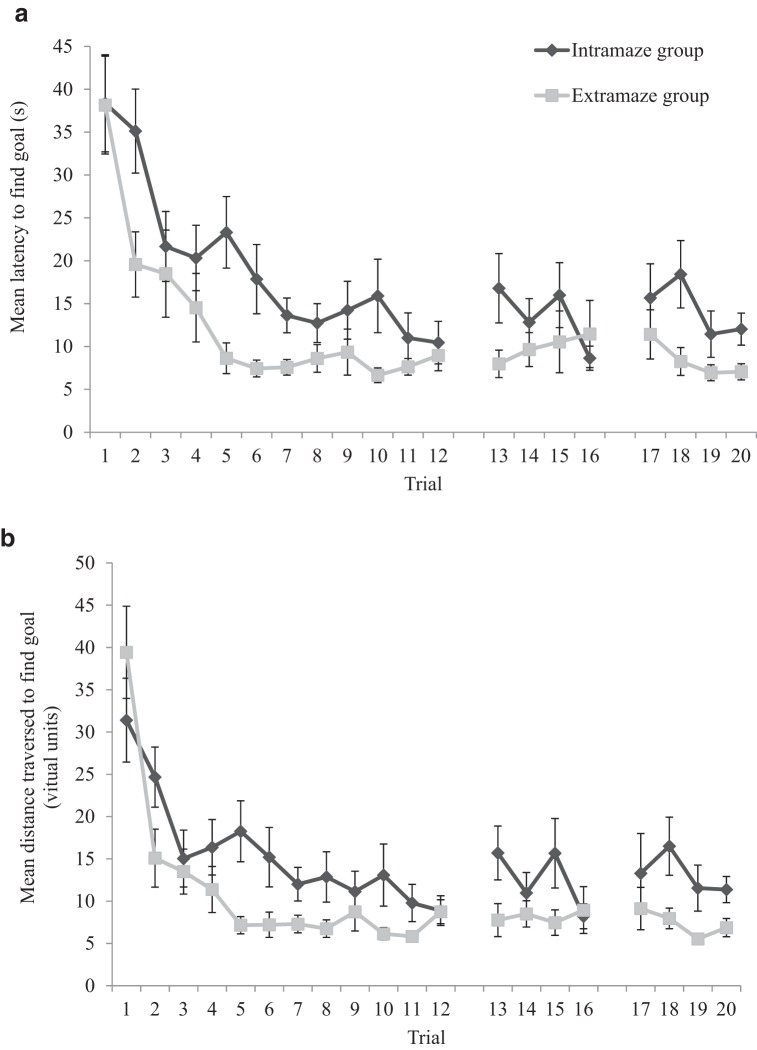
Mean latency to find the hidden goal during acquisition in Experiment 1 for both the intramaze and the extramaze groups (a). Mean distance traversed to find the hidden goal during acquisition in Experiment 1 for both the intramaze and the extramaze groups (b). Error bars represent ±1 *SEM*.

Table 1 displays the number of participants per trial, in both intramaze and extramaze groups, who failed to locate the hidden goal prior to the white flag appearing at the goal location after 60 s. From Trial 6 onward, nearly all participants, in both groups, successfully navigated to the hidden goal within 60 s. As such, the experience of finding the goal location was similar for participants in both groups. Figure 2a shows the latency, in seconds, from the beginning of each trial to enter the region of the arena defined as the hidden goal during the 20 acquisition trials, for both the extramaze and the intramaze groups. Figure 2b shows the distance traversed, in virtual units, before the hidden goal was found during the acquisition trials of the experiment (also for both groups). Both the mean latencies and the mean distances traversed decreased across the acquisition trials and, to our surprise, the intramaze group was slower, and traversed greater distances, to find the goal than the extramaze group during the experiment.[Table-anchor tbl1][Fig-anchor fig2]

In the following analyses of covariance (ANCOVAs), it was necessary to mean-center our covariate of age: Conceptually, a between-subjects covariate should not affect tests pertaining to within-subjects factors. However, it has been noted that tests of within-subjects main effects are altered if the mean of a covariate differs from zero (see Delaney & Maxwell, 1981; Thomas et al., 2009). By mean-centering age (subtracting the group mean age from individual ages of participants), the mean of the covariate becomes zero. Importantly, rescaling age in this manner does not alter tests of the main effect, or interactions with, the covariate itself. A two-way ANCOVA was conducted on individual latencies to find the goal, with a between-subjects variable of group (extramaze or intramaze), a within-subjects variable of trial (1–20), and mean-centered age as a covariate. The statistical model was customized to assess if group or trial interacted with the age covariate. This analysis revealed a significant main effect of trial, *F*(19, 836) = 13.18, mean square error (*MSE*) = 184.82, *p* < .001, η_p_^2^ = .23, confirming that participants found the goal quicker during the acquisition trials as the experiment progressed. There was also a main effect of group, *F*(1, 44) = 7.37, *MSE* = 1,112.52, *p* < .01, η_p_^2^ = .14, confirming that the extramaze group found the goal quicker during the experiment than the intramaze group. The main effect of age was also significant, *F*(1, 44) = 12.17, *MSE* = 184.82, *p* < .005, η_p_^2^ = .22, and there was also a significant Trial × Age interaction, *F*(19, 836) = 1.90, *MSE* = 184.82, *p* < .05, η_p_^2^ = .04. Parameter estimates, generated from the ANCOVA, for performance on each trial individually, revealed that older children found the goal quicker than younger children on Trials 1, 3, 4, 11, 12, and 14–16 (*t*s > 2.03, *p*s < .05). On all remaining trials, age did not reliably predict the time taken by participants to find the goal. Finally, there was no significant Group × Trial, *F*(19, 836) = 1.33, *MSE* = 185.03, *p* = .16, η_p_^2^ = .03, Group × Age (*F* < 1), or three-way (*F* < 1) interaction.

An identical two-way ANCOVA conducted on individual distances traversed to find the goal revealed a significant main effect of trial, *F*(19, 836) = 11.43, *MSE* = 155.98, *p* < .001, η_p_^2^ = .21, confirming that participants traversed shorter distances to find the goal over time. There was also a main effect of group, *F*(1, 44) = 8.62, *MSE* = 593.18, *p* < .01, η_p_^2^ = .16, confirming that the extramaze group traversed shorter distances to find the goal during the experiment than the intramaze group. There was, however, no main effect of age, *F*(1, 44) = 2.29, *MSE* = 593.18, *p* = .14, η_p_^2^ = .05. Nor were there significant Trial × Group, *F*(19, 836) = 1.44, *MSE* = 155.98, *p* = .10, η_p_^2^ = .03, Trial × Age, *F* < 1), Group × Age (*F* < 1), or a three-way, *F*(19, 836) = 1.19, *MSE* = 155.98, *p* = .26, η_p_^2^ = .03, interactions.

#### Test trials

**Figure 3 fig3:**
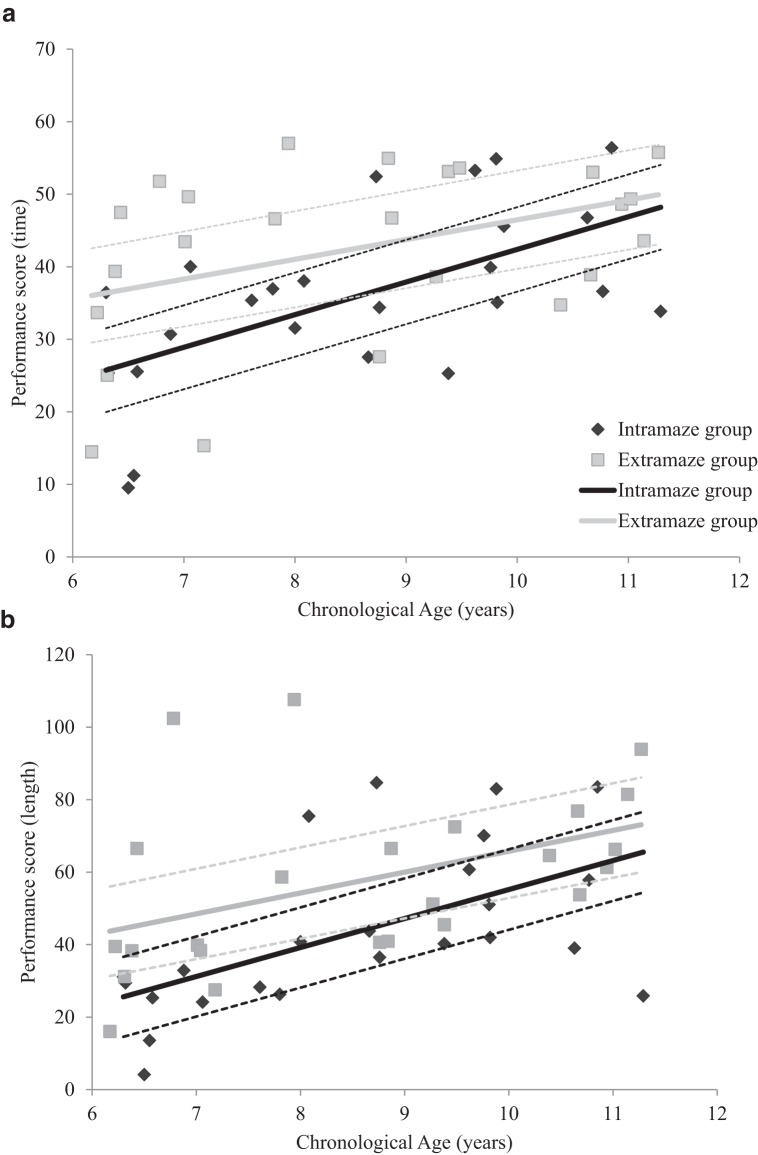
Performance scores for time (a) and path length (b) of individual participants plotted against the age of individual participants in both the extramaze and the intramaze groups of Experiment 1. Solid lines represent linear regression models of age predicting performance scores. Dotted lines represent both the upper and lower 95% confidence intervals of the regression models.

To analyze the data from the test trials, we divided the circular arena into four equal quadrants. For the intramaze group, we were interested in the amount of time spent, and distance traversed, in the quadrant that contained the intramaze landmark. As such, we designated the quadrant containing the landmark as the correct quadrant. Likewise, for the extramaze group, we were interested in the amount of time spent, or distance traversed, in the quadrant adjacent to the extramaze cue that was closest to the hidden goal, and so we designated this quadrant as the correct quadrant. To assess time spent in the correct quadrant in each group, we calculated a performance score that reflected time spent in this quadrant relative to one of the remaining three quadrants of the arena (i.e., those that did not contain the intramaze or extramaze cue of interest). For each test trial, one of the three remaining quadrants was designated as an incorrect quadrant, and the amount of time spent in this incorrect quadrant was subtracted from the time spent in the correct quadrant. As such, larger positive scores represent more time (or distance traversed) in the correct quadrant of the arena. The quadrant that was to the left of, the right of, or opposite the correct quadrant was assigned to be the incorrect quadrant an equal number of times. Measuring performance in this manner ensured that the way in which navigational behavior was compared during the test trials of Experiments 1−3 was equivalent. As will be seen later, in Experiments 2 and 3, the amount of time spent (or the distance traversed) is compared between two quadrants. Consequently, by performing the same calculations in Experiment 1, we are better able to make cross-experiment comparisons. Figure 3 displays individual performance scores plotted against age for both the intramaze and the extramaze groups. Older children spent more time, and traversed a greater distance, in the correct quadrant of the arena than did younger children.[Fig-anchor fig3]

To assess whether navigational performance was related to the age of participants, individual ages were regressed onto individual performance scores for each group separately. Following Thomas et al. (2009), individual ages were rescaled to reflect the months from the youngest age (MYA) tested within each group. Rescaling the age variable in this manner had no effect on the predictive ability of age in the regression model, instead, rescaling ages in such a manner adjusted regression coefficients such that the *y* intercept occurred at the youngest age tested within our sample. Hierarchical regression analyses were conducted on both time and path length performance scores for both groups separately. Individual MYAs were entered into the model first, after which BPVS raw scores, SAS scores, SWAN Inattentive subscale scores, and SWAN Hyperactive−Impulsive subscale scores were entered. As can be seen in Table 2, age reliably predicted (*p* < .05) time and path length performance scores in both the intramaze and the extramaze groups, with older children spending more time in the correct quadrant of the arena than younger children. Measures of receptive vocabulary, ASD, and ADHD did not make a significant contribution to either the regression model predicting time or the path length performance scores (see Table 3), although the measure of receptive vocabulary approached significance.[Table-anchor tbl2][Table-anchor tbl3][Table-anchor tbl4]

As previously stated, we wanted to compare the trajectories of children’s ability to navigate with intra- or extramaze cues in isolation. In particular, because we wanted to track the potential developmental onset of a bias toward using extramaze cues during navigation, we were interested whether older children in the extramaze group displayed better test performance than older children in the intramaze group. In the following statistical analysis, therefore, we rescaled individual ages of participants in the intra- and extramaze groups to reflect individual months from the oldest age (MOA) tested. Extrapolating regressions beyond the measured age range has poor validity and, therefore, comparing trajectories should be conducted at a point where the ages of participants within trajectories overlap. As such, we used 135.24 months as the zero point when rescaling individual ages of participants in the intra- and extragroups. To compare both the slope and the intercepts of the trajectories of the two groups, we performed a mixed-design univariate ANCOVA on both time and path length performance scores. Group (intramaze or extramaze) was entered as a between-subjects factor and individual MOAs were entered as a within-subjects covariate. The statistical model was again customized to assess the Group × MOAs interaction as well as the two main effects (see Thomas et al., 2009). By rescaling age to MOAs, a main effect of group would indicate a difference in the performance scores at the oldest overlapping age in both groups, while a significant interaction term would indicate a difference in the trajectory through younger ages between the extra- and intramaze groups. For both time, *F*(1, 44) = 15.38, *MSE* = 113.50, *p* < .001, η_p_^2^ = .26, and path length performance scores, *F*(1, 44) = 15.38, *MSE* = 411.55, *p* < .001, η_p_^2^ = .26, age reliably predicted performance but neither the main effect of group nor the Group × Age interaction was significant (*F*s < 1, η_p_^2^ < .03).^1^

### Discussion

During acquisition, older children took less time, and traversed a shorter distance, to find the hidden goal than younger children in both the intra- and the extramaze groups. At test, for both groups, older children spent more time, or traversed a greater distance, in the appropriate quadrant of the arena than did younger children. These data align with previous navigation studies conducted with children, in so much as older children displayed better navigational performance than younger children (e.g., Laurance et al., 2003). The most surprising result of Experiment 1, however, was that children in the extramaze group found the hidden goal quicker than children in the intramaze group during acquisition. Data from the test trials revealed no difference between the extra- and the intramaze groups, and no interaction between these factors and age, although, at least numerically, children’s performance scores were superior in the extramaze group than in the intramaze group.

The finding that children did not differ between groups on our task does not permit the conclusion that no difference exists. The absence of any difference in the developmental trajectories of using distal and proximal cues could be a consequence of a lack of sensitivity at test, especially given that we employed a between-subjects design. Moreover, children were tested when learning had reached an asymptotic level and, as such, the behavior observed at test, for both groups, could have been hindered by ceiling levels of performance. This suggestion gains a measure of support from the observation that children in the extra- and intramaze groups did differ during acquisition. To address these issues, Experiment 2 employed a within-subjects design during which children were trained to find a hidden goal that was signaled by a conjunction of extra- and intramaze cues. At test, the configuration of the extramaze cues was rotated, thus placing the response that each cue type elicited into conflict. This manipulation should ensure performance is not at ceiling level.

## Experiment 2

Experiment 2 assessed learning to extra- and intramaze cues when both of these cue types were present during acquisition. In this experiment, participants were, again, required to navigate to a hidden goal that remained in a constant position, in a circular arena; however, for *all* participants in Experiment 2, the arena contained an intramaze landmark and was surrounded by the four extramaze cues used in Experiment 1. During three test trials, the hidden goal was again removed and the extramaze cues were moved relative to the intramaze landmark—thus placing these navigation cues into conflict with each other. As such, it was possible to assess any cue preference children would display. On the basis of previous research (e.g., Bullens et al., 2010), we expected children older than 7 to show an adult-like bias toward searching near the extramaze cue that was closest to the goal location.

### Method

#### Participants

A total of 60 children (24 female), ages 63.00 (i.e., 5 years) to 141.72 (i.e., 11 years) months (*M* = 103.63 months, *SD* = 18.64), were again recruited during Summer Scientist Week. All children had normal or corrected-to-normal vision, and participated with full parental consent. In exchange for participation, children were given a token that allowed them to play a fairground game at the event. Measures of language ability (BPVS III *M*_raw_ = 111.69, *SD* = 24.11), ASD (SAS *M*_total_ = 25.09, *SD* = 6.99), and ADHD (SWAN Inattentive subscale *M*_total_ = −7.60, *SD* = 9.03; SWAN Hyperactive−Impulsive subscale *M*_total_ = −8.10, *SD* = 8.46) were routinely taken for children attending the event. Again, the ages of male (*M* = 8.39 years, *SD* = 1.55) and female (*M* = 8.95 years, *SD* = 1.64) participants did not differ statistically, *t*(58) = 1.36, *p* = .18.

#### Materials

The instruction arena used in Experiment 2 was identical to that outlined for Experiment 1. The experiment arena used for Experiment 2 was identical to the arena used in the extramaze group in Experiment 1, but, in addition, it also contained the same intramaze landmark that was used for the intramaze arena of Experiment 1. As before, the hidden goal was a square-shaped region (2.12 m × 2.12 m, invisible to participants), the center of which was located 2.62 m from both the intramaze landmark and one of the extramaze objects. As with Experiment 1, the extramaze cue that signaled the goal location was counterbalanced across participants such that each of the four extramaze cues signaled the goal location for 15 participants.

#### Procedure

The instructions given to children were identical to Experiment 1. Here, we describe only the procedural details of the current experiment.

##### Experiment

The procedure of the acquisition stage of Experiment 2 was identical to Experiment 1, thus, during acquisition, the hidden goal remained in the same location on each trial, which was equidistant from both the intramaze landmark and one of the four extramaze cues, the identity of which was counterbalanced within the group. For all test trials, which occurred following the 12th, 16th, and 20th acquisition trials, the hidden goal was removed, and participants were allowed to search for 60 s having, again, begun the trial in the center of the arena facing a random direction. Importantly, the extra- and intramaze cues that previously signaled the goal location during acquisition trials were placed into conflict. This was achieved by rotating the configuration of the extramaze cues at test, such that the intramaze landmark was next to a different extramaze cue on each of the three tests. Rotating the extramaze cues one, two, or three positions clockwise produced three test-trial arenas for each participant (see Figure 1). The order in which the three tests were presented was counterbalanced across the experiment such that each test arena was administered as the first, second, or third test equally often during the experiment. All remaining details pertaining to the experiment were identical to Experiment 1.

### Results

#### Acquisition

**Figure 4 fig4:**
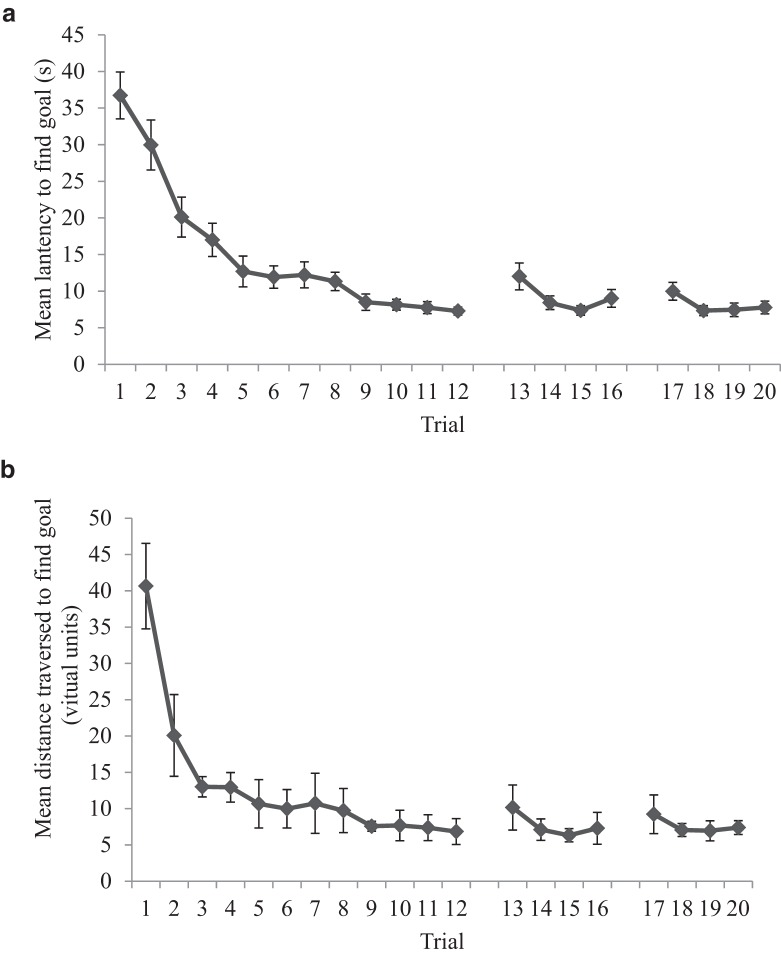
Mean latencies to find the hidden goal during the acquisition stage of Experiment 2 (a). Mean distances traversed to find the hidden goal during the acquisition stage of Experiment 2 (b). Error bars represent ±1 *SEM*.

Table 1 displays the number of participants, per trial, who failed to locate the hidden goal prior to the white flag appearing at the goal location after 60 s. From Trial 6 onward, nearly all participants successfully navigated to the hidden goal within 60 s, thus, the experience of finding the goal location was similar for participants in the experiment. Figure 4a shows the latency, in seconds, from the beginning of each trial to enter the region of the arena defined as the hidden goal during the 20 acquisition trials of the experiment. Figure 4b shows the distance traversed, in virtual units, to find the hidden goal during the acquisition trials of the experiment. Both the mean latencies and the mean distances traversed decreased across the acquisition trials. A one-way ANCOVA conducted on individual latencies to find the goal, with a within-subjects variable of trial (1–20) and covariate of mean-centered age, confirmed that participants found the goal quicker during the acquisition trials, *F*(19, 1,102) = 24.30, *MSE* = 154.91, *p* < .001, η_p_^2^ = .30. Age reliably predicted performance during the acquisition trials, *F*(1, 58) = 6.70, *MSE* = 487.00, *p* < .05, η_p_^2^ = .10, and there was a significant Trial × Age interaction, *F*(1, 1,102) = 1.74, *MSE* = 154.91, *p* < .05, η_p_^2^ = .03. Parameter estimates, generated from the ANCOVA, for performance on each trial individually revealed that older children found the goal quicker than younger children on Trials 1, 12, 13, and 16 (*t*s > 2.04, *p*s < .05). On all remaining trials, age did not reliably predict the time taken by participants to find the goal.[Fig-anchor fig4]

An identical ANCOVA conducted on individual distances traversed to find the goal revealed that participants traveled shorter distances to find the goal across the acquisition trials, *F*(19, 1,102) = 24.15, *MSE* = 147.12, *p* < .001, η_p_^2^ = .29. However, age did not significantly predict distances traversed during the acquisition trials, *F*(1, 58) = 2.32, *MSE* = 285.55, *p* = .13, η_p_^2^ = .04, nor was there a significant Trial × Age interaction, *F*(1, 1,102) = 1.32, *MSE* = 1,747.12, *p* = .16, η_p_^2^ = .02.

#### Test trials

**Figure 5 fig5:**
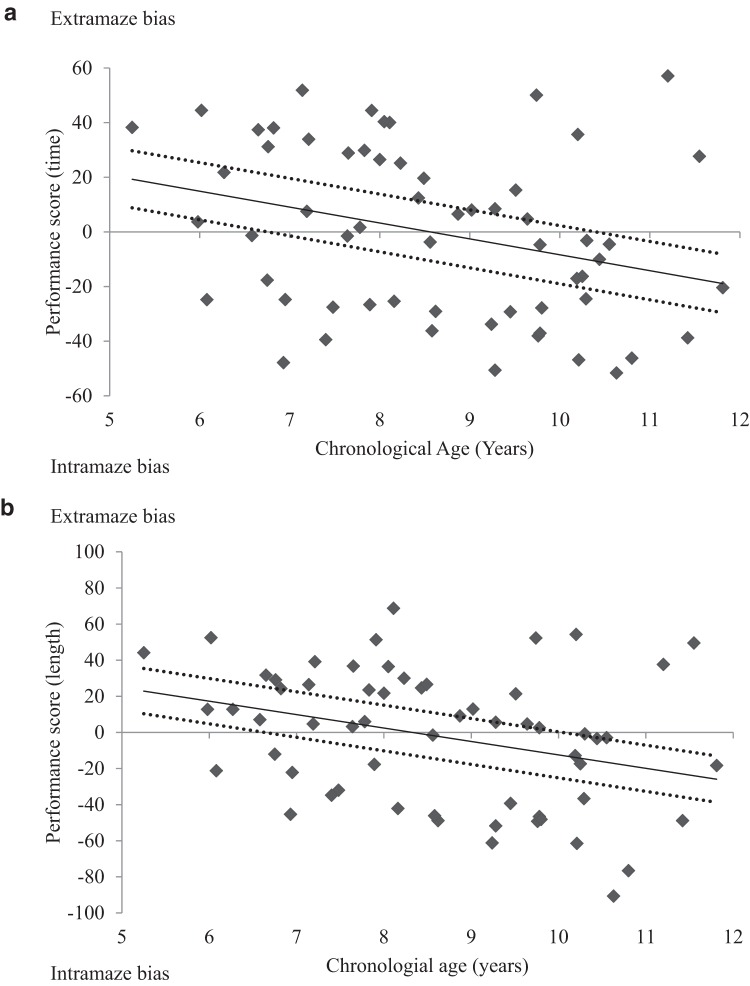
Performance scores for time (a) and path length (b) of individual participants plotted against the age of individual participants. The solid line represents the linear regression model of age predicting performance scores. Dotted lines represent the upper and lower 95% confidence intervals of the regression model.

The data from the test trials were analyzed in an identical manner to that in Experiment 1, however, rather than two quadrants being identified as correct and incorrect, they were now identified as intramaze and extramaze. Thus, the circular arena was divided into four equal quadrants, and the mean time spent and distance traversed in the quadrant that was (a) occupied by the intramaze landmark cue and (b) adjacent to the extramaze cue that was closest to the hidden goal during acquisition across the three tests was measured. To assess whether the age of participants influenced their search behavior on the test trials, we again created a test performance score for each child, which was the average time in the extramaze quadrant across the three test trials, minus the average time in the intramaze quadrant across the three test trials. The same calculation was also conducted with individual distances traversed within the extramaze and intramaze quadrants. This yielded performance scores where more positive values represented an increasing preference for searching in the extramaze quadrant and more negative values represented an increasing preference for searching in the intramaze quadrant during the test trials. Figure 5 displays individual test performance scores, for both time and path length measures, plotted against age for each participant. Younger children showed a bias toward searching near the extramaze cue during test trials, whereas older children were more likely to show a bias toward searching near the intramaze cue during test trials. To assess whether navigational performance was related to the age of participants, individual ages were regressed onto individual performance scores.^1^ Individual ages were again rescaled to reflect MYA within our sample. Hierarchical regression analyses were conducted on both time and path length performance scores, in which individual MYAs were entered into the model first, after which BPVS raw scores, SAS scores, SWAN Inattentive subscale scores, and SWAN Hyperactive−Impulsive subscale scores were entered. As displayed in Table 2, for time and path length data, age significantly predicted test trial performance scores (*p*s < .05), with older children spending more time, or traversing greater distances, in the intramaze quadrant than in the extramaze quadrant.^2^ The measures of receptive vocabulary, ASD and ADHD did not make a significant contribution to the regression model predicting either time or path length performance score (see Table 3).[Fig-anchor fig5]

### Discussion

Over the test trials in Experiment 2, during which the extramaze cues were placed into conflict with the intramaze landmark, younger children displayed a bias toward using the extramaze cues—spending more time searching, or traversing a greater distance, in the extramaze quadrant relative to the intramaze quadrant. In contrast, older children displayed a bias toward using the intramaze landmark at test, thus, searching for more time, and traversing a greater distance, in the intramaze quadrant relative to the extramaze quadrant.

In both virtual (Laurance et al., 2003) and real-world (Lehnung et al., 1998; Lehnung et al., 2003; Leplow et al., 2003; Overman, Pate, Moore, & Peuster, 1996) experiments, it has been found that children are able to navigate effectively on the basis of proximal landmarks before they are able to navigate effectively on the basis of distal information provided by the extramaze cues of an environment. In the experiment conducted by Bullens et al. (2010), during which intra- and extramaze cues were placed into conflict, children ages 5 or 7 displayed no bias toward navigating on the basis of either cue. Adults, however, displayed a bias toward navigating on the basis of extramaze cues. In light of these previous experiments, and particularly that by Bullens et al., we expected older children in our sample to display an adult-like bias toward searching near the extramaze cue during test trials. Instead, the opposite pattern of performance was found: Older children were biased toward searching near the intramaze cue at test.

Although measures of time and path length are linked, especially because all children moved around the virtual arena at a constant speed (cf. swim speed of rats; Bast et al., 2009), the two measures did reveal a subtle difference in behavior—in particular for the data from the acquisition stage of the experiment. For the time measure, during acquisition, age reliably predicted performance on certain trials. In contrast, for distance traversed to find the goal, age did not predict performance. As such, children of all ages traveled the same distance to find the goal location on a given acquisition trial. The time measure included any behavior performed on a given trial, including rotations around the *y* axis that were used to bring different objects into view. In contrast, the path length measure only included movement in the *x* and *z* planes when traveling to the goal location. One possible explanation of this discrepancy between the measures is that the age-related difference in time to find the goal reflects a difference in the time it took for younger children to *decide* which part of the arena to navigate to, compared with older children.

Standard associative theories of learning (e.g., Mackintosh, 1975; Rescorla & Wagner, 1972; Pearce & Hall, 1980) have recently been applied to the study of navigational learning (e.g., Miller & Shettleworth, 2007, 2008; Pearce, 2009) and may help to reconcile the results of Experiment 2 with the absence of any difference, at test, in Experiment 1—if it is assumed that the salience of extra- and intramaze cues varies with age. For example, in our arenas, the extramaze cues may have been more salient than the intramaze cue for young children, whereas the intramaze cue may have been more salient than the extramaze cues for older children. According to standard associative theories, when two cues are trained in isolation, as they were in Experiment 1, the differential salience of cues will influence the rate at which learning proceeds, but not the ultimate asymptotic performance. As such, both cues will eventually acquire the same associative strength and, thus, control behavior equivalently. In contrast, any differential salience of the two cues will always affect the relative salience of the two cues when they are trained in compound, as was done in Experiment 2. When trained in compound, the more salient cue will overshadow learning about the less salient cue (Mackintosh, 1976; Miles & Jenkins, 1973), thus, the more salient cue will acquire greater associative strength and, ultimately, therefore, exert greater control over behavior than the less salient cue. Where this analysis is incomplete, however, is that if the extramaze cues were more salient than the intramaze landmark for younger children, and vice versa for older children, then this should have been evident in the analysis of the effects of age on acquisition performance in the intra- and extramaze groups of the acquisition data of Experiment 1. The source of this absence of an effect from Experiment 1 remains to be determined.

The important finding from Experiment 2 was that older children displayed a bias toward using the intramaze cue at test. It is, however, somewhat difficult to interpret this result. An adulthood preference towards extramaze information, over intramaze information, has mainly been observed in overshadowing paradigms in which participants are trained with a compound of distal and proximal cues and subsequently tested with only one of these cues (e.g., Doeller & Burgess, 2008). Although there are reports of adults displaying a preference for extramaze, over intramaze, cues when the two cue types are placed into conflict in real-world experiments (e.g., Bullens et al., 2010), thus far, we have no evidence whether adults would display such a bias in our virtual environment. Experiment 3 was, therefore, conducted to assess whether adults would show any cue preference in the present environment.

## Experiment 3

The purpose of Experiment 3 was to ascertain whether adults would display a bias toward using extramaze over intramaze cues, and to assess the extent to which each cue type in isolation would control adult navigational behavior. A compound group was trained and tested in the same arena as children were in Experiment 2. Thus, adults were trained to find a hidden goal in a circular arena that contained an intramaze landmark and that was surrounded by four extramaze cues. At test, the hidden goal was removed from the arena, and the extramaze cues were placed into conflict with the intramaze landmark, again, in the same manner as in Experiment 2. Following the results presented by Bullens et al. (2010), it was expected that adults would show a bias toward navigating in the quadrant of the arena adjacent to the extramaze cue that was closest to the hidden goal during acquisition, compared with the quadrant that contained the intramaze cue. Two additional groups were included that replicated the conditions of Experiment 1. In the extramaze group, adults were trained to find a hidden goal in a circular environment that was orientated by four extramaze cues, but that did not contain an intramaze landmark. Participants were then given test trials in which the hidden goal was removed. Finally, in the intramaze group, adults were trained to find a hidden goal that contained an intramaze landmark, but was not orientated by the four extramaze cues. Again, test trials, in which the hidden goal was removed, were administered.

### Method

#### Participants

A total of 72 undergraduate students (26 female) ages 18 to 29 years (*M* = 19.61 years, *SD* = 2.34) were recruited from the student population of the University of Nottingham. All participants had normal or corrected-to-normal vision, and were randomly allocated to one of the three conditions of the experiment. On completion of the experiment, participants were given course credit or £5.

#### Materials

All material details for the compound group were the same as outlined for Experiment 2, and all material details for the extra- and intramaze groups were identical to those outlined in Experiment 1. Adults sat at a standard table to complete the experiment.

#### Procedure

All procedural details were identical to those outlined for Experiments 1 and 2.

### Results

#### Acquisition

**Figure 6 fig6:**
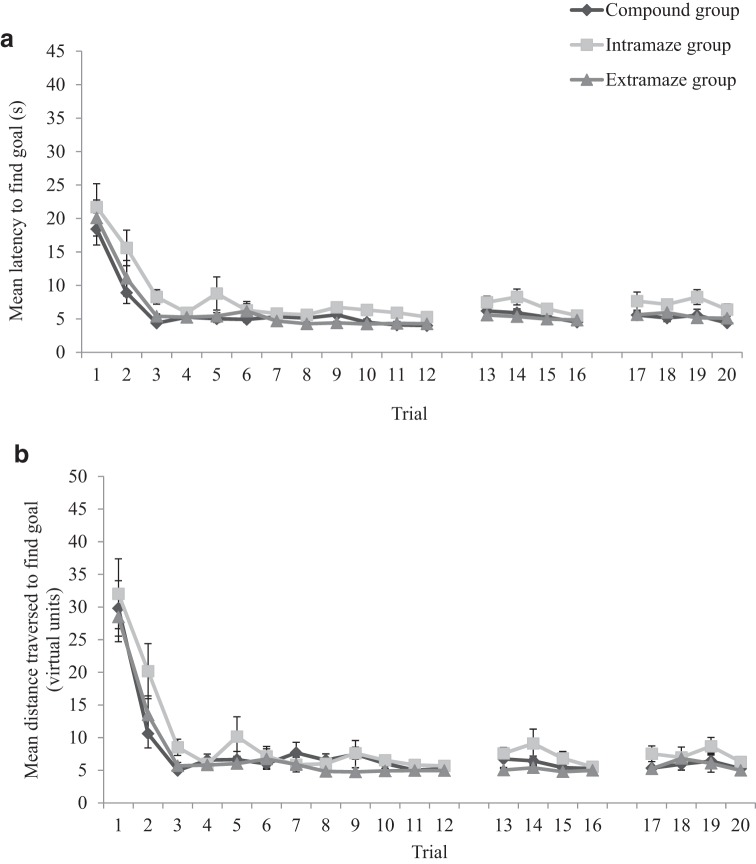
Mean latencies to find the hidden goal during acquisition in Experiment 3 for the compound, intramaze, and extramaze groups (a). Mean distances traversed to find the hidden goal during acquisition in Experiment 3 for the compound, intramaze, and extramaze groups (b). Error bars represent ±1 *SEM*.

Figure 6a shows the latency, in seconds, from the beginning of each trial to enter the region of the arena defined as the hidden goal during the 20 acquisition trials for the compound, extramaze, and intramaze groups. Figure 6b shows the distance traversed, in virtual units, to find the hidden goal during the acquisition trials of the experiment, again for all three groups of the experiment. Mean latencies and distances traversed to find the goal decreased during the experiment, and the intramaze group took longer, and traversed a greater distance, to find the goal compared with the compound and extramaze groups. A two-way analysis of variance (ANOVA) of individual latencies to find the goal, with a between-subjects variable of group (compound, extramaze, or intramaze) and a within-subjects variable of trial (1–20) revealed a significant main effect of trial, *F*(19, 1,311) = 32.21, *MSE* = 27.42, *p* < .001, η_p_^2^ = .32, confirming that participants found the goal quicker as the experiment progressed. There was also a significant effect of group, *F*(2, 69) = 9.87, *MSE* = 63.84, *p* < .001, η_p_^2^ = .22. Bonferroni-corrected *t* tests conducted on individual mean acquisition times revealed that the intramaze group was significantly slower to find the goal than the compound, *t*(46) = 3.79, *p* < .001, and extramaze, *t*(46) = 3.65, *p* < .005, groups, but that there was no difference between the latter two groups (*t* < 1). There was no significant Group × Trial interaction (*F* < 1, η_p_^2^ = .02).[Fig-anchor fig6]

An identical two-way ANOVA conducted on individual distances traversed to find the goal revealed comparable results. There was a significant main effect of trial, *F*(19, 1,311) = 40.04, *MSE* = 56.76, *p* < .001, η_p_^2^ = .37, indicating that participants traversed significantly shorter distances to find the goal location during the experiment. There was also a significant main effect of group, *F*(2, 69) = 5.80, *MSE* = 91.75, *p* < .01, η_p_^2^ = .14. Post hoc, Bonferroni-corrected, *t* tests conducted on individual mean distances traversed during acquisition trials revealed the intramaze group traversed significantly greater distances to find the goal than both the compound, *t*(46) = 2.31, *p* < .05, and extramaze, *t*(46) = 3.28, *p* < .005, groups, but again there was no difference between the compound and extramaze group (*t* < 1). Again, there was no Trial × Group interaction (*F* < 1, η_p_^2^ = .02).

#### Test trials

**Figure 7 fig7:**
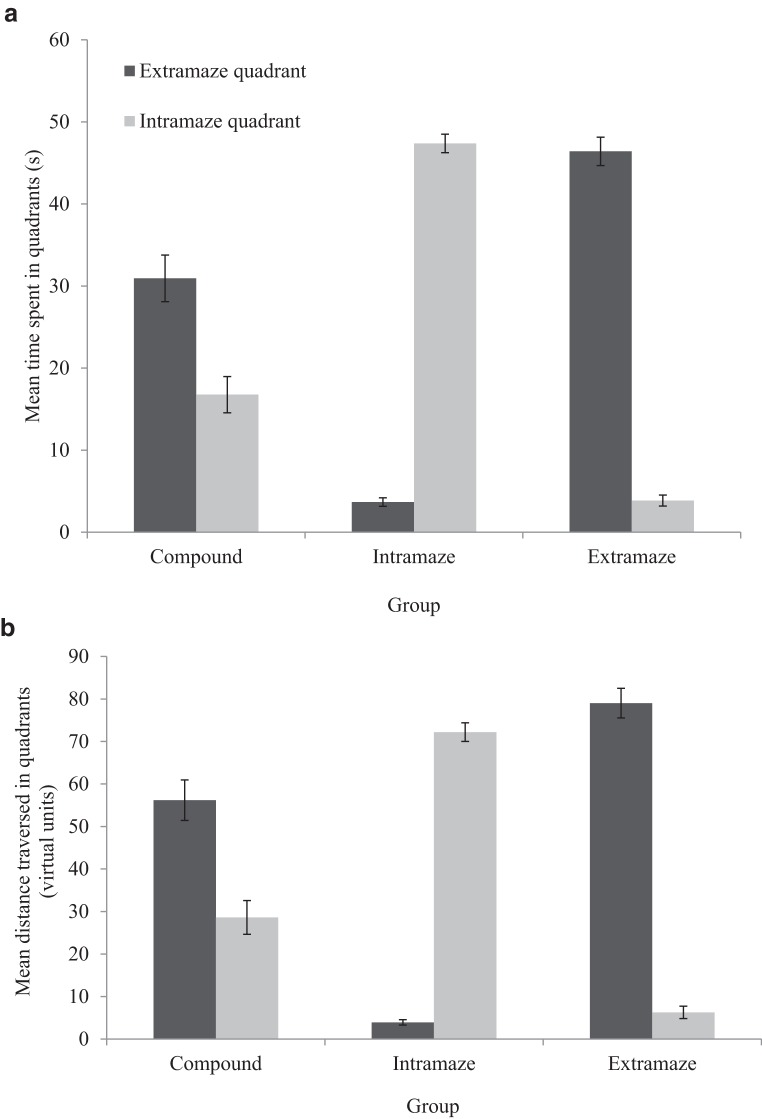
Mean time spent in quadrants over all three test trials of Experiment 3 for the compound, intramaze, and extramaze groups (a). Mean distance traversed in quadrants over all three test trials of Experiment 3 for the compound, intramaze, and extramaze (b). Error bars represent ±1 *SEM*.

To analyze the test trial data, the circular arena was divided into four equal quadrants, and the mean time spent and distance traversed in the quadrant occupied by the intramaze landmark and extramaze cue across the three tests was calculated for the compound group. Data from the extra- and intramaze groups in the current experiment were analyzed in the same manner as described for Experiment 1. Thus one of the three quadrants that did not contain the relevant extramaze or intramaze cue, respectively, was designated as an incorrect quadrant (see Results section, Experiment 1). Importantly, the relative locations of the correct and incorrect quadrants in the extramaze and intramaze groups were matched to the relative locations of the extramaze and intramaze quadrants for the compound group. Figure 7a displays the time spent in the extra- and intramaze quadrants of the arena collapsed across the three test trials. Figure 7b shows the distance traversed in the extra- and intramaze quadrants collapsed across the three test trials of the experiment. Consistent with the data collected from adults by Bullens et al. (2010), the compound group displayed a preference, at test, for searching near the extramaze cue, both in terms of the time spent and distance traversed, relative to the intramaze cue. As would be expected, the extramaze and intramaze groups displayed a preference for searching in the extramaze or intramaze quadrants, respectively. Finally, the time spent by the compound group in the extra- and intramaze quadrants, during all three tests, was much shorter than the time spent in those quadrants in the extra- and intramaze groups, respectively.[Fig-anchor fig7]

A two-way ANOVA conducted on individual time spent in quadrants, with a between-subjects variable of group (compound, extramaze, or intramaze) and a within-subjects variable of quadrant (extramaze or intramaze), revealed no main effect of group, *F*(2, 69) = 2.30, *MSE* = 16.11, *p* = .11, η_p_^2^ = .06. There was, however, a main effect of quadrant, *F*(1, 69) = 5.34, *MSE* = 127.24, *p* < .05, η_p_^2^ = .07, and a significant Quadrant × Group interaction, *F*(2, 69) = 182.25, *MSE* = 127.24, *p* < .001, η_p_^2^ = .84. Simple main effects analysis showed that, within the extramaze group, participants spent significantly more time searching in the (correct) extramaze quadrant relative to the (incorrect) intramaze quadrant, *F*(1, 69) = 170.82, *p* < .001, η_p_^2^ = .71. Within the intramaze group, participants spent significantly more time searching in the (correct) intramaze quadrant than the (incorrect) extramaze quadrant, *F*(1, 69) = 180.07, *p* < .001, η_p_^2^ = .72. Finally, and most importantly, participants in the compound group spent significantly more time searching in the extramaze quadrant than they did in the intramaze quadrant, *F*(1, 69) = 18.95, *p* < .001, η_p_^2^ = .22. Between-subjects *t* tests revealed that participants in the compound group spent less time searching in the extramaze quadrant than participants in the extramaze group, *t*(46) = 4.65, *p* < .001, and, likewise, participants in the compound group spent less time searching in the intramaze quadrant than participants in the intramaze group, *t*(46) = 9.44, *p* < .001.

An identical ANOVA, conducted on individual distances traversed in quadrants, revealed the same effects: There were main effects of group, *F*(2, 69) = 3.14, *MSE* = 362.45, *p* < .05, η_p_^2^ = .08, quadrant, *F*(1, 69) = 11.32, *MSE* = 362.45 *p* < .001, η_p_^2^ = .14, and a significant Quadrant × Group interaction, *F*(2, 69) = 171.70, *p* < .001, η_p_^2^ = .83. Simple main effects analysis again showed that, within groups, the compound, *F*(1, 69) = 25.18, *p* < .001, η_p_^2^ = .27, and extramaze, *F*(1, 69) = 175.16, *p* < .001, η_p_^2^ = .72, groups traversed a greater distance in the extramaze quadrant than the intramaze quadrant, whereas the intramaze group traversed a greater distance in the intramaze quadrant than the extramaze quadrant, *F*(1, 69) = 154.38, *p* < .001, η_p_^2^ = .69. Between-groups *t* tests again revealed that the compound group traversed shorter distances in the extramaze quadrant relative to the extramaze group, *t*(46) = 3.86, *p* < .001, and shorter distances in the intramaze quadrant relative to intramaze group, *t*(46) = 9.62, *p* < .001.

### Discussion

When the two cue domains were put into conflict, adults preferentially searched by the extramaze cue that previously signaled the goal location, rather than by the intramaze landmark that also previously signaled the goal location. This was revealed by participants in the compound group spending more time, or traversing a greater distance, in the extramaze quadrant at test than in the intramaze quadrant. These data support previous findings addressing adult cue preference in similar paradigms, such as those of Doeller and Burgess (2008) and Bullens et al. (2010). This result was obtained with the same arena in which children were tested in Experiment 2, where it was found that older (but not younger) children preferentially searched near the intramaze landmark. Consequently, it appears that the adult preference for extramaze information does not necessarily reflect a gradual, ordinal, shift toward a distal preference during childhood. Importantly, these results were obtained where differences in both physical and numeric floor effects were avoided. The use of a virtual environment meant that the real-world height differences of adults and children were unlikely to have affected the results and, furthermore, the adults and children were both tested following training to a comparable (i.e., high) level.

## General Discussion

During the acquisition stage of Experiment 1, children navigating on the basis of extramaze cues found the hidden goal more efficiently than children navigating on the basis of intramaze cues. Once acquisition was complete, however, we could no longer detect this bias in a series of test trials. Experiment 2 demonstrated that when extra- and intramaze cues, which had together previously signaled a goal location, were placed in conflict, older children were more likely to search near the intramaze cue than near the extramaze cue, and vice versa for younger children. Finally, Experiment 3 showed that adults, navigating in the same virtual arenas as the children, displayed a preference for using information provided by the extramaze cues because they spent significantly longer searching near the extramaze cue at test than the intramaze cue. It is worth reiterating that the virtual navigation task controlled for the eye height of participants, ensuring that both the extra- and the intramaze cues, as well as the circular wall, appeared at the same height for both adults and children.

The data from Experiment 3 are in keeping with the real-world experiment conducted by Bullens et al. (2010). However, our examination of children’s individual performance during the test trials in Experiment 2 does not support their proposal that, during navigation, children may rely weakly on both proximal cues within the search environment and distal cues outside (Bullens et al., 2010). The 95% confidence intervals of Figure 5 suggest that, between the ages of 7 and 10, a shift from a bias toward using extramaze cues to the use of intramaze cues develops. Given that older children were expected to exhibit a preference toward searching near the extramaze cue, akin to adult participants, this result was surprising. In light of this, an appropriate avenue of future research would be to test adolescents to pinpoint when a reliable bias toward using distal information develops in navigational behavior.

Previous navigation experiments have suggested that the ability to navigate on the basis of proximal landmarks develops before the ability to navigate on the basis of extramaze information (e.g., Lehnung et al., 1998; Lehnung et al., 2003; Leplow et al., 2003), or that children may rely weakly on both proximal and distal information during navigational tasks (Bullens et al., 2010). The data from our experiment, however, suggest that young children are able to navigate on the basis of distal information and, moreover, may preferentially use distal cues, over proximal landmark cues, to guide navigation at a young age. In previous experiments, children have been required to either sequentially navigate to a number of baited light points (e.g., Lehnung et al., 1998), or to remember the location of two objects (Bullens et al., 2010), in circular environments that were orientated by extramaze cues and that contained intramaze landmarks. One notable difference between these previous tasks and the experiments reported here is their relative complexity: In our experiments, children were only required to learn a single goal location. Given less challenging tasks demands, it appears that young children are able to navigate on the basis of distal landmark cues. It is, however, worth noting that the majority of previous experiments exploring this issue have been conducted in real-world environments, whereas our task was conducted in a virtual environment. Although Laurance et al. (2003) observed that children effectively navigated a two-dimensional screen display as if it were three-dimensional space, it is still the case that the sensory input entering the navigational system differs in virtual reality experiments compared with real-world experiments. For instance, participants receive vestibular, proprioceptive, and somesthetic inputs during real-world experiments, but not in virtual reality experiments (Banta Lavenex & Lavenex, 2010). Additional research will be required to ascertain whether differences in task complexity, navigational input, or other variables contribute to determining the circumstances under which young children are able to navigate on the basis of distal information.

To avoid conflating effects observed with *boundary information* (e.g., Doeller & Burgess, 2008), and the effects observed here (and by Bullens et al., 2010) with *extramaze cues*, it is worth noting the differences between the three experiments. First, the extramaze cues used in the current experiments, and those conducted by Bullens et al. (2010), deviate from those used by Doeller and Burgess (2008), because the distal landmarks were placed just beyond the walls of the environment. In contrast, boundary information in the task described by Doeller and Burgess was provided by a circular enclosure that was orientated by cues rendered at an infinite distance. As such, the extramaze cues in our experiment, and those used by Bullens et al. (2010), provided positional information that could be used to localize a goal position, whereas the distal landmarks in the study by Doeller and Burgess (2008) could not be used to localize a specific position. Second, the experiments presented here, and by Doeller and Burgess, were conducted using a virtual arena, while the experiment conducted by Bullens et al. was conducted within a real-world setting. Third, in the experiments reported here, we measured the time spent and the distance traversed within quadrants of the search environment, whereas Bullens et al. and Doeller and Burgess measured error distance when relocating an object. A pertinent point here, though, is that, despite these differences, the three studies converge on the finding that adults preferentially use extramaze (or distal) information over intramaze (or proximate) landmark cues. Thus, the way in which intra- and extramaze information is used during navigation is comparable, despite substantially different task demands and environments.

Children in the intramaze group of Experiment 1 were significantly slower at finding the goal location during acquisition relative to children in the extramaze group. For adult participants in Experiment 3, the intramaze group was also significantly slower at finding the goal location during acquisition trials relative to both the compound and the extramaze groups. These data are consistent with previous findings that an array of landmarks supports a narrower area of search than a single landmark (e.g., Della Chiesa, Pecchia, Tommasi, & Vallortigara, 2006). It has been suggested that, when navigating in rich environments, multiple vectors from several surfaces can define a goal location (Mou & Zhou, 2013). In sparse environments, which contain fewer surfaces, there are fewer vectors that can define a target location. Consequently, rich environments support a more precise localization of the goal area, and ultimately more efficient navigation, relative to sparse environments. In Experiments 1 and 3, the multiple cues that were present in the extramaze and compound groups may have supported better localization of the hidden goal than did the single landmark in the intramaze group, thus, participants in the compound and extramaze groups were able to find the hidden goal quicker than participants in the intramaze group. Experiments that are being conducted in our laboratory are currently testing this possibility by replicating the current experiments but under circumstances in which the number of extramaze cues match the number of intramaze landmarks.

The precise reason why children, at least up to the age of 11, develop a bias toward using intramaze landmark information over extramaze information remains to be determined. One framework that potentially permits an understanding of our pattern of results is the overlapping waves theory (Siegler, 1996), which is based on three assumptions: (a) children do not use a single method to solve a problem, but use a variety of strategies, (b) the variety of strategies used by children to solve a problem coexist over lengthy periods of time, and, finally, (c) experience changes the degree to which children will rely on given strategies, as well as allowing for new strategies to be formed. The model states that older strategies may cease to be used and, moreover, the model permits the use of preexisting strategies even in the presence of later developing strategies that might be more useful to a given problem. In terms of our data from Experiment 2, the overlapping waves theory could appeal to the possibility that young children were more likely to use a strategy that was reliant on the extramaze cues. Children in the middle of our age range were likely to choose either an intra- or an extramaze strategy, giving the appearance of no cue preference; and older children in our sample were more likely to use a strategy that was reliant on intramaze landmark cues. As has been mentioned before, it will be necessary to assess navigational behavior of adolescent participants to determine the developmental period for which a proximal landmark strategy is likely to be used over the adult-like distal strategy that supersedes this initial landmark strategy, and perhaps more interestingly, to identify experiential events that may coincide with these shifts.

Finally, it is worth noting that, although age was a significant predictor of performance scores in our task, the regression models accounted for a relatively small proportion of the variance in our measures. This suggests that individual differences, other than age, may influence the navigational behavior of children. That said, the measures of language ability, attention, and social abilities (relating to autism) taken for children made no significant contribution to predicting performance scores on our task. These measures were, however, opportunistic in nature, and as such might not have been sensitive enough to correlate with performance on our task. For example, it might be expected that spatial relational grammar (e.g., “to the left of . . .”) would correlate with navigational performance better than the more general measure of receptive vocabulary used here (see Hermer-Vazquez et al., 2001). Other cognitive abilities, however, might be more likely to influence navigational behavior, such as spatial working memory, which has been shown to correlate with performance on navigational tasks (e.g., Castelli, Latini Corazzini, & Geminiani, 2008; Pellicano et al., 2011).

### Conclusion

In a virtual navigation task, adults displayed a preference for navigating to a hidden goal on the basis of extra- rather than intramaze cues. Interestingly, older children in our sample (who were expected to show an adult-like bias toward extramaze cues) were more likely than younger children to show a preference toward searching near an intramaze landmark at test. This suggests the bias toward using extramaze information that is observed in adulthood is a late developing trait and, moreover, one that supersedes a preference for navigating with intramaze landmarks during development. A fruitful avenue of future research would therefore be to assess the point during development at which a reliable bias toward using extramaze information occurs to fully account for the development of adult-like navigational abilities. More generally, the fact that navigational behavior appears to continue developing during the adolescent years provides further rationale for assessing the maturation of adult-like behaviors in adolescence, and the continual development of the adolescent brain (see Blakemore & Choudhury, 2006).

## Figures and Tables

**Table 1 tbl1:** The Number of Participants, per Acquisition Trial, Who Failed to Find the Hidden Goal Within 60 s in the Intramaze and Extramaze Groups of Experiment 1, in the Sample Recruited for Experiment 2, and in the Compound, Intramaze, and Extramaze Groups of Experiment 3

Trial	1	2	3	4	5	6	7	8	9	10	11	12	13	14	15	16	17	18	19	20
Experiment 1																				
Extramaze group	8	2	2	2	0	0	0	0	1	0	0	0	0	0	2	1	1	0	0	0
Intramaze group	6	3	2	2	3	1	0	0	1	2	1	0	2	0	1	0	3	2	1	0
Experiment 2	15	12	6	4	2	0	2	0	0	0	0	0	3	0	0	1	1	0	0	0
Experiment 3																				
Extramaze group	1	1	0	0	0	0	0	0	0	0	0	0	0	0	0	0	0	0	0	0
Intramaze group	2	1	0	0	1	0	0	0	0	0	0	0	0	0	0	0	0	0	0	0
Compound group	0	0	0	0	0	0	0	0	0	0	0	0	0	0	0	0	0	0	0	0

**Table 2 tbl2:** Multiple Linear Regression Models: Predicting Test Trial Performance Scores Obtained From Experiment 1 and 2 by Participant Age

Predicting variable	*B*	*SE* *B*	β	*p*
Experiment 1				
Extramaze group time				
Constant	36.04	3.89		
Age	.23	.11	.409	.047
			*R*^2^ = .17	
Length				
Constant	43.69	7.40		
Age	.48	.21	.445	.029
			*R*^2^ = .20	
Intramaze group time				
Constant	−25.75	3.55		
Age	−.38	.11	−.598	.002
			*R*^2^ = .36	
Length				
Constant	−25.61	6.77		
Age	−.67	.20	−.571	.004
			*R*^2^ = .33	
Experiment 2				
Time				
Constant	19.27	9.01		
Age	−.48	.20	−.30	.020
			*R*^2^ = .09	
Path length				
Constant	22.87	10.79		
Age	−.62	.24	−.32	.013
			*R*^2^ = .10	

**Table 3 tbl3:** Multiple Linear Regression Models: Predicting Test Trial Performance Scores Obtained From Experiments 1 and 2 by Individual Difference Measures

Predicting variable	Correlation coefficient	*p*
Experiment 1		
Extramaze group time		
BPVS raw scores	−.10	.65
ADHD: Inattentive total	.02	.94
ADHD: Hyperactive−Impulsive total	.30	.18
SAS total	−.36	.11
Length		
BPVS raw scores	−.38	.08
ADHD: Inattentive total	.17	.44
ADHD: Hyperactive−Impulsive total	.24	.27
SAS total	−.13	.57
Intramaze group time		
BPVS raw scores	.30	.18
ADHD: Inattentive total	−.05	.83
ADHD: Hyperactive−Impulsive total	−.03	.91
SAS total	−.24	.28
Length		
BPVS raw scores	.39	.07
ADHD: Inattentive total	−.02	.91
ADHD: Hyperactive−Impulsive total	.11	.63
SAS total	−.25	.25
Experiment 2		
Time		
BPVS raw scores	.18	.39
ADHD: Inattentive total	.08	.54
ADHD: Hyperactive−Impulsive total	.08	.55
SAS total	−.04	.76
Length		
BPVS raw scores	.12	.40
ADHD: Inattentive total	.12	.39
ADHD: Hyperactive−Impulsive total	.13	.33
SAS total	−.05	.70
*Note*. ADHD = attention-deficit/hyperactivity disorder; BPVS = British Picture Vocabulary Scale; SAS = Social Aptitudes Scale.		

**Table 4 tbl4:** Multiple Linear Regression Model: Predicting Test Trial Performance Scores, Obtained Only From Test 3 of Experiment 2, by Participant Age

Predicting variable	*B*	*SE* *B*	β	*p*
Time				
Constant	22.15	9.31		
Age	−.56	.21	−.33	.009
			*R*^2^ = .11	
Path length				
Constant	29.99	12.09		
Age	−.79	.27	−.35	.006
			*R*^2^ = .12	

## Footnotes

1 Although the ages of male and female participants did not differ in either the extramaze or the intramaze groups of Experiment 1(and they also did not differ in the sample recruited for Experiment 2), it is possible that that gender could interact with age, such that the navigational behavior of males and females would follow different trajectories. To explore this possibility, we treated individual time and path length performance scores, from each group separately, with a univariate analysis of covariance in which gender was entered as a between-subjects factor and age was entered as a covariate. In none of these statistical analyses did gender interact with age.

2 A potential objection to this analysis is that if the navigational biases of children of different ages are to be compared, then the task difficulty should be equivalent for the different ages studied. Because young children were slower to find the hidden goal during earlier acquisition trials than older children, there is a possibility that that the differences we observed may be due to task difficulty rather than age per se. However, this shortcoming can be circumnavigated by examining spatial biases at a point on the acquisition curve where the different ages do not differ. At this point, task difficulty, at least as measured by task performance, is equated. This can be achieved by replicating this analysis but only on data drawn from the final test session; by this point in training, parameter estimates from the analysis of covariance indicated no effect of age on task performance. Hierarchical regression analyses of performance scores calculated only from the third test trial showed that age significantly predicted time and path length performance scores (see Table 4). As before, older children were predicted to spend more time, or to traverse a greater distance, in the intramaze quadrant than in the extramaze quadrant, despite being at an equivalently (high) point on the overall task.
